# High Monopolar Spindle 1 Is Associated with Short Survival of Cholangiocarcinoma Patients and Enhances the Progression Via AKT and STAT3 Signaling Pathways

**DOI:** 10.3390/biomedicines9010068

**Published:** 2021-01-13

**Authors:** Piya Prajumwongs, Ratthaphong Phumphu, Orawan Waenphimai, Worachart Lert-itthiporn, Kulthida Vaeteewoottacharn, Sopit Wongkham, Yaovalux Chamgramol, Chawalit Pairojkul, Kanlayanee Sawanyawisuth

**Affiliations:** 1 Department of Biochemistry, Faculty of Medicine, Khon Kaen University, Khon Kaen 40002, Thailand; prajumwongspiya@gmail.com (P.P.); ratthaphong_ph@kkumail.com (R.P.); w_orawan@kkumail.com (O.W.); woracle@kku.ac.th (W.L.-i.); kulthidava@kku.ac.th (K.V.); sopit@kku.ac.th (S.W.); 2 Cholangiocarcinoma Research Institute, Khon Kaen University, Khon Kaen 40002, Thailand; cyaova@yahoo.com (Y.C.); chawpa@kku.ac.th (C.P.); 3 Department of Pathology, Faculty of Medicine, Khon Kaen University, Khon Kaen 40002, Thailand

**Keywords:** MPS1, Cholangiocarcinoma, migration, invasion, AKT, STAT3, EMT, MMPs

## Abstract

Cholangiocarcinoma (CCA) is a malignancy of the bile duct epithelium. The major problems of this cancer are late diagnosis and a high rate of metastasis. CCA patients in advanced stages have poor survival and cannot be cured with surgery. Therefore, targeting molecules involved in the metastatic process may be an effective CCA treatment. Monopolar spindle 1 (MPS1) is a kinase protein that controls the spindle assemble checkpoint in mitosis. It is overexpressed in proliferating cells and various cancers. The functional roles of MPS1 in CCA progression have not been investigated. The aims of this study were to examine the roles and molecular mechanisms of MPS1 in CCA progression. Immunohistochemistry results showed that MPS1 was up-regulated in carcinogenesis of CCA in a hamster model, and positive expression of MPS1 in human CCA tissues was correlated to short survival of CCA patients (*n* = 185). Small interfering RNA (siRNA)-induced knockdown of MPS1 expression reduced cell proliferation via G2/M arrest, colony formation, migration, and invasion. Moreover, MPS1 controlled epithelial to mesenchymal transition (EMT)-mediated migration via AKT and STAT3 signaling transductions. MPS1 was also involved in MMPs-dependent invasion of CCA cell lines. The current research highlights for the first time that MPS1 has an essential role in promoting the progression of CCA via AKT and STAT3 signaling pathways and could be an attractive target for metastatic CCA treatment.

## 1. Introduction

Cholangiocarcinoma (CCA), a primary cancer of bile duct epithelial cells, is associated with infection by the carcinogenic liver fluke (*Opisthorchis viverrini*, Ov), which is endemic in the Northeastern region of Thailand [1]. CCA has a high mortality rate according to its high rate of metastasis, which is due to difficulties with early diagnosis [2]. Treatment of CCA patients with metastasis by surgery or chemotherapy is rarely successful [3,4]. Therefore, inhibiting molecules involved in cell progression or metastasis could be an alternative strategy for treatment of metastatic CCA.

Monopolar spindle 1 (MPS1), or TTK (Thr/Tyr kinase), is an essential protein in the spindle assembly checkpoint (SAC) during mitosis [5]. MPS1 acts in several aspects of mitotic checkpoint control including mitotic SAC activation, recruitment of SAC components to kinetochores, centrosome duplication, error correction of kinetochore-microtubule attachment, and chromosome alignment in mitosis [5,6,7]. MPS1 is highly expressed in proliferating cells such as bone marrow, stem cells, and cancers [8]. Overexpression of MPS1 has been reported in breast, colon, glioblastoma, liver, and pancreatic cancers [9,10,11,12,13]. Multiple functions of MPS1 are involved in neoplastic progression including the cell survival and metastatic potential of bladder, liver, lung, and breast cancers [14,15,16,17].

Overexpression of MPS1 in CCA tissues has been published previously [18], however the clinical impact and the functional roles of MPS1 in CCA progression have not been explored. In the current study, the expression of MPS1 was assessed in hamster and human CCA tissues using immunohistochemistry to reveal the clinical relevance of increased MPS1 expression. The functional roles and underlying mechanisms of MPS1 in the progression of CCA, including cell proliferation, migration, and invasion, were evaluated in CCA cell lines.

## 2. Experimental Section

### 2.1. Datasets of MPS1 Expression from Public Database

The mRNA expression levels of *MPS1* (*TTK*) in CCA samples were retrieved from two databases. First, we analyzed CCA data from The Cancer Genome Atlas Program (TCGA) via GEPIA website (http://gepia.cancer-pku.cn/) [19]. Second, Gene Expression Omnibus (GEO) database (https://www.ncbi.nlm.nih.gov/geo/) was used to access GSE89749 [20]. The GEO data were obtained by GEOquery package [21] and normalized by lumi package [22] in R version 3.6.1. (R core Team, Vienna, Austria). Patients were divided into 2 groups using the median of *MPS1* expression level as a cut-off. Overall survival of patients based on *MPS1* expression was also analyzed.

### 2.2. Hamster Liver tissues and CCA Patient Tissues

Paraffin-embedded liver tissues obtained from *Opisthorchis viverrini* (Ov)-associated CCA hamster model (*n* = 60). The experimental design was divided into four groups of five hamsters including non-treated, Ov-infected, a potent carcinogen N-nitrosodimethylamine (NDMA; 12.5 ppm in water ad libitum)-treated, and Ov + NDMA treated groups as previously described [23]. Hamsters were sacrificed at 1-, 3-, and 6-months post-treatment. The protocol of the study was approved by the Ethics Committee for Animal Research, Khon Kaen University (AEMDKKU 001/2558, Date of approval 9 February 2015).

Paraffin-embedded liver tissues from 185 CCA patients were obtained from the specimen bank of the Cholangiocarcinoma Research Institute, Faculty of Medicine, Khon Kaen University. The protocol of collection and study were approved by Ethics Committee for Human Research, Khon Kaen University (HE591063, Date of approval 9 February 2016).

### 2.3. Cell Lines

CCA cell lines (KKU-055, KKU-100, KKU-213A and KKU-213B) were established as previously described [24,25] and obtained from the Japanese Collection of Research Bioresources Cell Bank (Osaka, Japan). An immortalized cholangiocyte cell line, MMNK1 was previously characterized [26]. Cells were cultured in Dulbecco’s Modified Eagle Medium (DMEM) supplemented with 10% fetal bovine serum (FBS) and a 1% antibiotic-antimycotic (Gibco, Thermo Fisher Scientific, Grand Island, NY, USA). Cells were incubated at 37 °C with 5% CO_2_ in a humidified incubator.

### 2.4. Immunohistochemistry (IHC)

To determine the expression of MPS1 in human and hamster CCA tissues. The IHC staining was performed using standard protocol. Briefly, the paraffin-embedded liver tissues were deparaffinized in xylene and rehydrated in ethanol. Antigens were retrieved by autoclaving in Tris-EDTA buffer pH 9.0 for 3 min. The slides were incubated with 1:600 mouse monoclonal anti-MPS1 (EMD Millipore, Darmstadt, Germany) overnight at 4 °C followed by incubation with secondary antibody. Immunoreactivity was detected by addition of 3, 3-diaminobenzidine substrate solution. Sections were counterstained with Mayer’s hematoxylin. The positive cytoplasmic staining of MPS1 was evaluated using H-score, which was calculated from intensity and percentage of positively stained tumor cells [27]. The intensity of immunostaining was assigned one of four scores as follows: 0; no immunostaining, 1+; weak intensity, 2+; moderate intensity and 3+; strong intensity. Calculation of H-score using the formula: H-score = [(3 × percentage of strongly staining immunopositive cells) + (2 × percentage of moderately staining immunopositive cells) + (1 × percentage of weakly staining immunopositive cells)], giving a range of 0–300 [27]. H-scores were evaluated by two separate assessors. The median H-score of CCA tissues was used as the cut-off value to divide into negative and positive MPS1 expression cases.

### 2.5. Western Blot Analysis

Cell lysates were prepared and Western blot was performed as previously described [28]. Proteins were detected by specific antibodies including AKT, phospho-AKT (s473), STAT3, phospho-STAT3 (Y705), Claudin-1, Slug, Vimentin (Cell Signaling Technology, Danvers, MA, USA), and MPS1 (BD biosciences, San Jose, CA, USA) antibodies. GAPDH (EMD Millipore, Darmstadt, Germany) was used as an internal control. The immunoreactivity was detected by ECL^TM^ Prime Western Blotting Detection (GE Healthcare, Buckinghamshire, UK). The chemiluminescent signal was captured by ImageQuant LAS 4000 mini-image analyzer and signal intensities were analyzed by ImageQuant^TM^ TL analysis software (GE Healthcare, Buckinghamshire, UK).

### 2.6. Knockdown MPS1 Gene by siRNA

Pooled siRNA (mixture of 2 different siRNAs; *MPS1*-453: 5’ GCACGTGACTACTTTCAAA 3’ and *MPS1*-1868: 5’ TCCGACTTTATGATTATGAAA 3’) were used to silence the expression of *MPS1* in CCA cell lines. CCA cells were transfected with siMPS1 (si) or scramble control (sc) using Lipofectamine 2000 reagent (Invitrogen, Carlsbad, CA, USA) according to the manufacturer’s protocols. Cells were harvested at the indicated times for further analysis.

### 2.7. Cell Proliferation Assay (MTT Assay) 

CCA cells (1500 cells) were seeded into 96-well plates and transfected with siMPS1 or scramble control (sc) for 24, 48, 72, and 96 h. MTT reagent (3-(4,5-dimethylthiazolyl-2)-2,5-diphenyltetrazolium bromide) was added into the wells to obtain 0.5 mg/mL final concentration (Sigma-Aldrich, Darmstadt, Germany). After 4 h incubation, DMSO was added to dissolve the insoluble formazan complex and the absorbance at 540 nm was measured by microplate reader.

### 2.8. Colony Formation Assay

One hundred cells were seeded into 6-well plates and cultured for 10 days. Cells were fixed with 4% (*w/v*) paraformaldehyde in PBS for 20 min and stained with 0.5% (*w/v*) crystal violet in methanol for 20 min. The number of colonies containing at least 50 cells were counted under microscope.

### 2.9. Cell Cycle Analysis

CCA cells were seeded at a density of 1 × 10^5^ cells into 6 cm cell culture dishes and transfected with silencers for 72 and 96 h. Cells were fixed in 70% ethanol at 4 °C overnight, stained with 10 μg/mL propidium iodide (PI), and analyzed using a BD LSR II™ flow cytometer. Data analysis was performed using FlowJo™ software.

### 2.10. Cell Migration and Invasion Assay

Cell migration assay was performed using an 8 µm pore size transwell insert (Corning Incorporated, Corning, NY, USA). MPS1 knockdown cells (50,000 cells) in serum free media were added into the upper chamber of transwell insert. DMEM with 10% FBS was used as a chemoattractant in the bottom chamber. After incubation at 37 °C for 33 h in KKU-055 and 9 h in KKU-213A, cells in the upper chamber were removed. The inserts were fixed with 4% paraformaldehyde and stained with 0.4% sulforhodamine B in 0.1% acetic acid. The number of migrated cells were counted from five microscopic fields/ insert. For the invasion assay, the transwell inserts were coated with 40 µg of Matrigel^TM^ (BD biosciences, San Jose, CA, USA) before use. The number of invaded cells were analyzed as stated for the migration assay. Incubation times were 36 h in KKU-055 and 12 h for KKU-213A.

### 2.11. Gelatin Zymography

Cells (50,000 cells) were seeded into 24-well plates and cultured in serum free media for 24 and 48 h. The conditioned media were collected and analyzed for the activity of MMPs on 10% polyacrylamide gel containing 0.1% gelatin. After protein separation, the gel was renatured with 2.5% Triton X-100 and incubated in a developing buffer (50 mM Tris PH 7.4, 5 mM CaCl_2_, 1 µM ZnCl_2_, 0.01% NaN_3_). Gel was stained with 0.5% Coomassie blue R250 for 1 h and de-stained until the appearance of a clear zone on the gel. The image was inverted to show dark bands on clear background. The relative fold change of enzyme activity was quantified and assigned the scramble (sc) control as 1.

### 2.12. Statistical Analysis

The differences of continuous data between two dependent groups were analyzed by either independent t-test (parametric test) or Mann–Whitney test (non-parametric test). Values are presented as the mean ± SD. Statistical comparisons between groups were tested using the Student’s t- test. *p* < 0.05 was considered as statistically significant. The data were analyzed by GraphPad Prism^®^ 7.0 software (GraphPad software, Inc., San Diego, CA, USA) and SPSS 23 software (SPSS, Chicago, IL, USA). The survival curve was created and analyzed using Kaplan-Meier estimate with Log-rank test.

## 3. Results

### 3.1. MPS1 Expression was Up-Regulated during Carcinogenesis of Hamster CCA 

**Figure 1:**
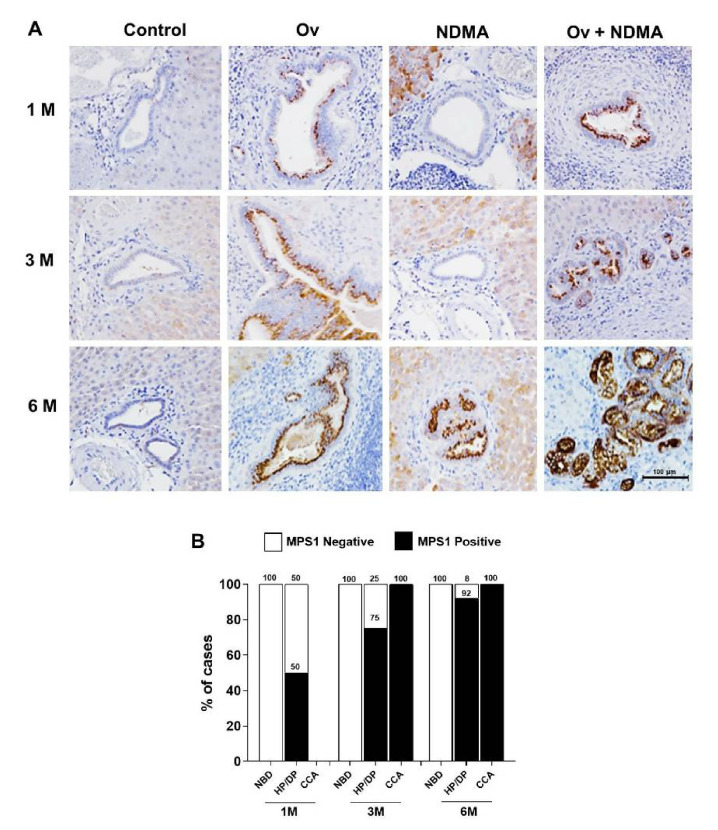
MPS1 is associated with cholangiocarcinogenesis in a hamster model. (**A**) Immunohistochemistry (IHC) staining of MPS1 in liver tissues from four groups of hamsters including control, carcinogen N-nitrosodimethylamine (NDMA)-treated, Ov-infected, and combination of NDMA + Ov at one, three, and six months after treatment. (**B**) Comparison of the percent of MPS1 positive cases between NBD, hyperplasia/dysplasia (HP/DP), and Cholangiocarcinoma (CCA). Median H-score of MPS1 in hamster CCA tissues was 70, which was used as a cut off value to categorize the samples into two groups; MPS1 negative and MPS1 positive.

To determine the role of MPS1 in cholangiocarcinogenesis in the hamster CCA model, expression of MPS1 was investigated in hamster liver tissues using immunohistochemistry. Figure 1A shows negative staining of MPS1 in normal bile ducts (NBD) in the control group and all treatment groups at every time point. A gradual increase in the number of MPS1 positive cases in hyperplasia/dysplasia (HP/DP) was found in Ov-infected group at one, three, and six months. MPS1 was highly expressed in HP/DP of NDMA treated group at six months. In the Ov + NDMA treatment, HP/DP with MPS1 positive staining were found at every time point. At three and six months, CCA had developed and all tissues were MPS1 positive (Table 1). The median H-score of MPS1 in hamster CCA tissues was 70, which was used as a cut off value to categorize the samples into MPS1 negative and MPS1 positive cases. The number of MPS1 positive cases in HP/DP gradually increased with time of treatment (Figure 1B). These results reveal that MPS1 could be detected early in precancerous lesions (HP/DP) and was highly expressed in CCA. This is the first report of MPS1 expression being up-regulated during cholangiocarcinogenesis.

### 3.2. High Expression of MPS1 was Associated with Poor Survival of CCA Patients

**Figure 2:**
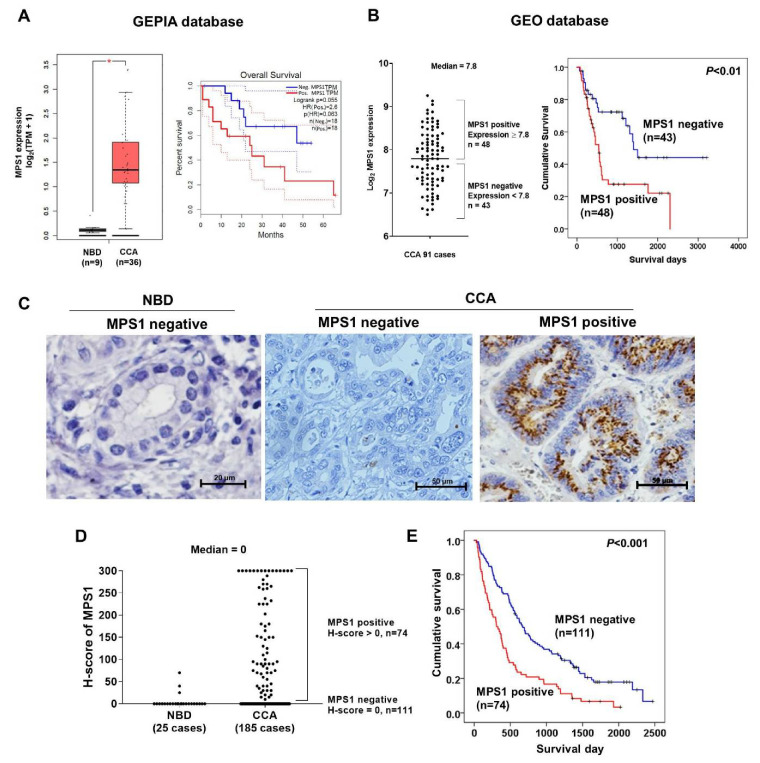
Overexpression of MPS1 or TTK in human CCA tissues. (**A**) A box plot of MPS1 transcript level in 36 human CCA tissues compared to 9 normal bile ducts (NBD). Comparison of overall survival curves between negative and positive MPS1 expressing patients. Data were retrieved from GEPIA database. TPM is transcript per million. * *p* < 0.05. (**B**) A dot plot of MPS1 transcript level in 91 human CCA patients categorized in two groups: MPS1 negative and MPS1 positive, using the median value of MPS1 expression (7.8). Cumulative survival times of MPS1 positive patients were significantly shorter than those of MPS1 negative patients (*p* < 0.01). Data were retrieved from GEO database. (**C**) Representative IHC staining of MPS1 protein in 185 CCA patient tissues. MPS1 expression was not detected in NBD (magnification 400×) and MPS1 negative CCA tissues (magnification 200×). MPS1 positive CCA showed cytoplasmic staining of MPS1 (magnification 200×). (**D**) Distribution of MPS1 expression in 25 NBD and 185 CCA. Median H-score was 0, which was used as a cut-off value for dividing the MPS1 positive and negative cases. (**E**) Cumulative survival times of MPS1 positive patients (*n* = 74) were significantly shorter than those of MPS1 negative patients (*n* = 111) *p* < 0.001.

MPS1 expression in human CCA tissues was retrieved from the GEPIA and GEO databases (GSE GSE89749). The MPS1 transcript levels of 36 CCA tissues and 9 normal tissues retrieved from the GEPIA database indicated that the expression level of MPS1 in CCA tissues was significantly higher than in normal tissues (Figure 2A, *p* < 0.05). CCA patients were divided according to the median MPS1 level into positive and negative expression groups and CCA patients with positive expression of MPS1 tended to have shorter survival time than those with negative expression (log-rank, *p* = 0.055). Additionally, the expression levels of MPS1 were used to categorize CCA patients (*n* = 91) from the GEO database into positive and negative MPS1 expression groups based on the median value of 7.8 (Figure 2B). MPS1 positive patients (*n* = 48) were correlated with shorter survival time than MPS1 negative patients (*n* = 43) (*p* < 0.01; Figure 2B). These results indicate that high expression of MPS1 correlates with poor survival of CCA patients. The characteristics of the patients from GEPIA and GEO databases are shown in Supplementary Table S1. Moreover, we examined the protein expression of MPS1 in tissue microarrays of 185 Ov-associated CCA patients using immunohistochemistry. MPS1 protein expression was mostly negative in adjacent normal bile ducts (NBD) while it was increased in CCA tissues (Figure 2C). The distribution of H-scores of MPS1 in NBD (*n* = 25) and CCA (*n* = 185) is shown in Figure 2D. The median H-score of 0 was used as a cut-off value to divide patients into MPS1 negative and positive groups. It showed that MPS1 expression was remarkably increased in CCA tissues. No significant correlation between MPS1 expression and clinicopathological features of CCA patients was revealed by univariate analysis (Supplementary Table S2).

In Figure 2E, CCA patients are divided into two groups as negative (111/185 cases, 60%) and positive staining for MPS1 (74/185 cases, 40%). The median survival time of patients with positive MPS1 expression was significantly shorter than those with negative MPS1 expression (*p* < 0.001). Cox regression analysis was performed to determine whether MPS1 expression indicates a risk factor of poor survival in CCA patients. Multivariate analysis revealed that the hazards ratio (HR) of patients who were classified as MPS1 positive was 2.015 (95% CI 1.442–3.073) compared to MPS1 negative cases. There was a significant association between MPS1 expression and survival, as shown in Table 2 (*p* < 0.001), which indicates that MPS1 is an independent prognostic factor for poor survival of CCA patients.

### 3.3. Suppression of MPS1 by siRNA Reduced Cell Proliferation, Colony Formation and Induced G2/M Arrest

**Figure 3:**
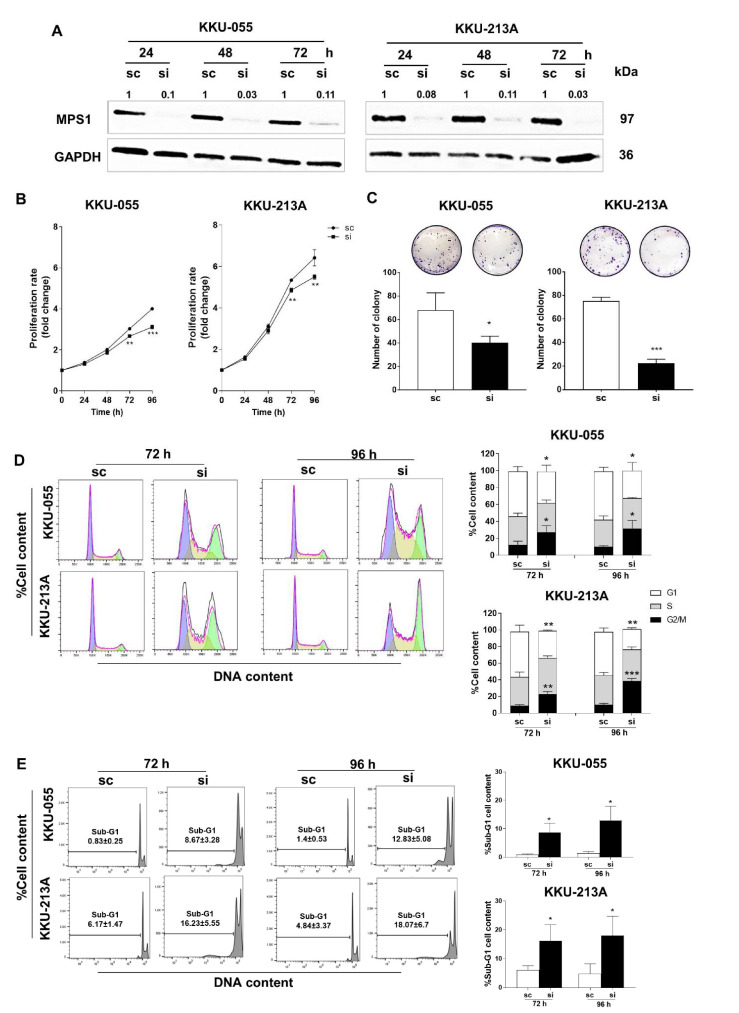
MPS1 contributed to cell proliferation, colony formation, and altered cell cycle of CCA cell lines. (**A**) Knockdown efficiency of MPS1 specific siRNA at 24 to 72 h in KKU-055 and KKU-213A cell lines. Quantification of each band was normalized to GAPDH and is shown as the numbers above the corresponding panels. (**B**) Cell proliferation rates of siMPS1 treated KKU-055 and KKU-213A were determined at 24 to 96 h. (**C**) Colony formation assay shows decreasing numbers of colonies after MPS1 knockdown in CCA cell lines. (**D**) Flow cytometry histograms demonstrate decreasing G1 phase and increasing G2/M arrest in siMPS1 treated cells at 72 and 96 h. (**E**) Sub-G1 populations were analyzed by flow cytometry. A time-dependent increase in the percentage of sub-G1 populations was observed upon MPS1 knockdown. Data are presented as mean ± SD of three independent experiments. * *p* < 0.05, ** *p* < 0.01 and *** *p* < 0.001.

The endogenous expression of MPS1 protein was measured in a panel of CCA cell lines using Western blot analysis (Supplementary Figure S1). KKU-055 and KKU-213A CCA cell lines were used to study the functions of MPS1 in the progression of CCA. MPS1 expression was transiently suppressed by siRNA for 24, 48 and 72 h in CCA cell lines. Western blot results showed that MPS1 specific siRNA (siMPS1) effectively suppressed MPS1 expression at 24 to 72 h in both CCA cell lines (Figure 3A).

To determine whether MPS1 is involved in CCA cell proliferation, MPS1 expression was silenced for 24 to 96 h and cell proliferation was measured by MTT assay. Suppression of MPS1 had no effect at 24 and 48 h but it significantly reduced cell proliferation at 72 and 96 h in both cell lines (Figure 3B). In addition, the colony formation assay demonstrated that numbers of colonies in siMPS1-treated cells were significantly lower than scramble control treated cells (* *p* < 0.05 for KKU-055 and *** *p* < 0.001 for KKU-213A), as shown in Figure 3C. Flow cytometry was performed to evaluate whether suppression of MPS1 affected cell cycle distribution. Figure 3D illustrates a marked decrease in the cell population in G1 phase while the cell population in G2/M arrest was significantly increased in siMPS1-treated KKU-055 and KKU-213A cell lines. Moreover, significant increases in the sub G1 population (apoptotic cells) were found in siMPS1-treated CCA cell lines at 72 and 96 h (Figure 3E). Western blot analysis confirmed that decreasing of Cyclin B1 (G2/M arrest), increasing of Bax pro-apoptotic and decreasing of Mcl-1 anti-apoptotic markers (apoptosis) after MPS1 knockdown in CCA cell lines (Supplementary Figure S2).

### 3.4. Knockdown of MPS1 Inhibited EMT-Mediated Migration via AKT and STAT3 Activation and Attenuated MMPs-Dependent Invasion.

**Figure 4:**
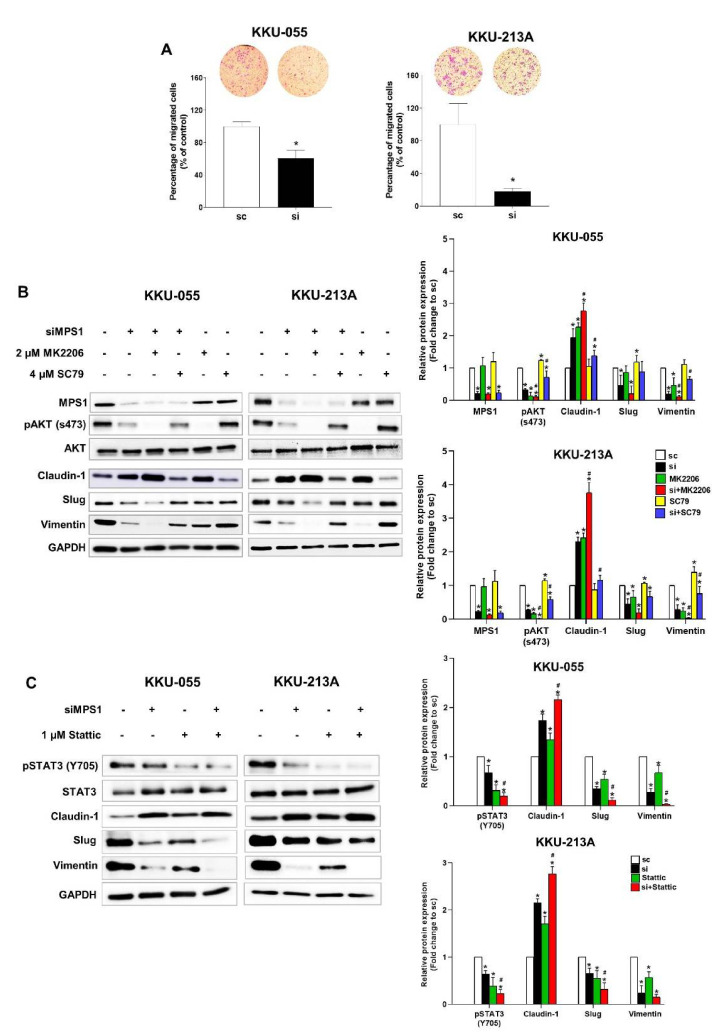
MPS1 promoted EMT-mediated migration via AKT and STAT3 signaling pathways. (**A**) Boyden chamber assay was performed to evaluate the migration of siMPS1 treated KKU-055 and KKU-213A cells. (**B**) Cells were pre-treated with siMPS1 and an AKT inhibitor (MK2206) or an AKT activator (SC79). The expression of EMT-related proteins was analyzed using Western blot assay. Representative results of claudin-1, slug, vimentin, pAKT (s473), and pSTAT3 (Y705) are shown. GAPDH was used as an internal control. Quantification of protein expression was normalized with GAPDH and by assigning the scramble (sc) control as 1. Bar graph shows the mean ± SD band intensities of proteins from three independent experiments. (**C**) Combination of siMPS1 and a STAT3. inhibitor (Stattic) greatly altered the expression of EMT-related markers in KKU-055 and KKU-213A cell lines. Protein loading and protein quantification were performed as described for Figure 4B. Significant difference (*p* < 0.05) when compared with sc (*) or si (^#^).

The contribution of MPS1 to cell migration was next investigated. Knockdown of MPS1 significantly decreased the migration of KKU-055 and KKU-213A when compared to scramble control (Figure 4A). EMT is the process involved in cell migration and the expression of epithelial marker (claudin-1), and mesenchymal markers (slug and vimentin) are regulated via the AKT signaling pathway. To examine whether alteration of the expression of EMT markers under MPS1 knockdown was regulated via activation of AKT signaling, KKU-055 and KKU-213A cells were treated with siMPS1 for 24 h and then either a specific inhibitor of AKT (2 µM MK2206) or a pan-AKT activator (4 µM SC79) for 24 h and subjected to Western blot analysis. The results show that either siMPS1 or MK2206 treatment significantly reduced phosphorylation of AKT (s473) and expression of slug and vimentin, but induced expression of claudin-1 when compared to untreated control (* *p* < 0.05) in both CCA cell lines. The combination of siMPS1 and MK2206 further reduced phosphorylation of AKT (s473), and slug and vimentin expression, while increasing claudin-1 expression when compared to siMPS1 alone (^#^ *p* < 0.05). Conversely, SC79 treatment of siMPS1 treated cells restored phosphorylation levels of AKT and increased slug and vimentin expression, while decreasing claudin-1 expression compared to siMPS1 treatment in both cell lines (Figure 4B).

MPS1- dependent regulation of EMT processes via the STAT3 signaling pathway was also examined. KKU-055 and KKU-213A cells were treated with siMPS1 for 24 h followed by a STAT3 inhibitor (1 µM Stattic) for 24 h and protein expression was analyzed by Western blot. It showed that siMPS1 or Stattic treatment significantly reduced the phosphorylation of STAT3 (Y705) and slug and vimentin expression, but induced claudin-1 expression when compared to control in both cell lines (* *p* < 0.05). Co-treatment with siMPS1 and Stattic significantly decreased the phosphorylation of STAT3 (Y705) and the expression of slug and vimentin. This combination also increased claudin-1 expression when compared to siMPS1 alone (^#^ *p* < 0.05), as shown in Figure 4C. Taken together, these results suggest that MPS1 controlled EMT-related molecules via activation of the AKT and STAT3 signaling pathways in CCA cells.

**Figure 5:**
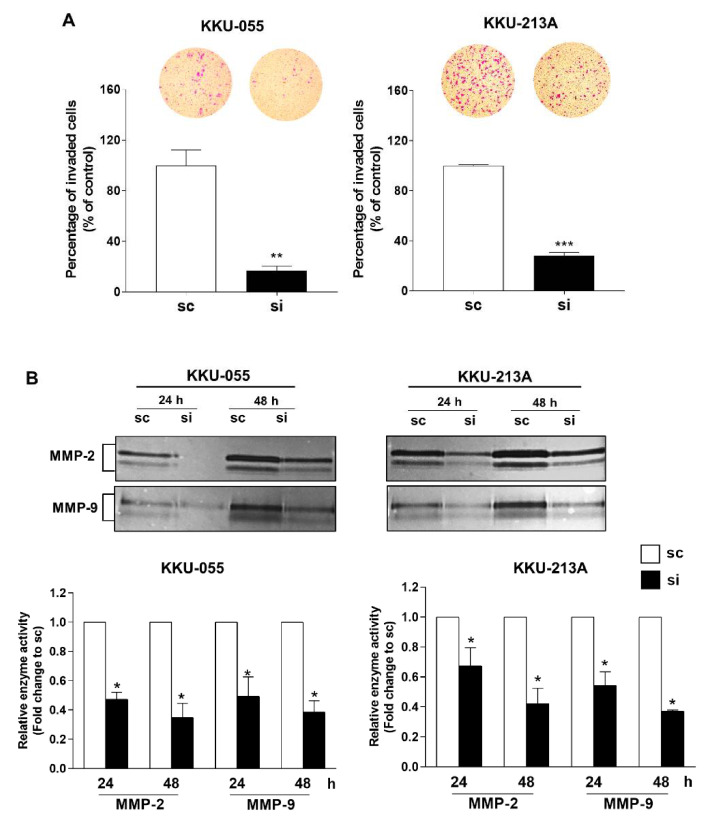
Suppression of MPS1 attenuated MMPs-dependent invasion in CCA cell lines. (**A**) The percentage of invaded cells after knocking down MPS1 in KKU-055 and KKU-213A cell lines. Results are mean ± SD of three independent experiments. (**B**) Gelatin zymogram of MMP-2 and MMP-9 activities in siMPS1-treated cells. The enzyme activity was relatively quantified to scramble (sc) control. Data are presented as mean ± SD from three independent experiments. * *p* < 0.05, ** *p* < 0.01 and *** *p* < 0.001.

Matrigel coated transwell inserts were used to study the invasive ability of MPS1 knockdown cells. MPS1 suppression significantly decreased the invasion of KKU-055 and KKU-213A cells, as shown in Figure 5A. The gelatin zymography assay demonstrated that the activities of MMP-2 and MMP-9 were significantly decreased in conditioned media from siMPS1 treated cells at 24 and 48 h when compared to the scramble control (Figure 5B). Our findings indicate that MPS1 promotes MMPs-dependent invasion of CCA cells.

## 4. Discussion

MPS1 or TTK protein controls the mitotic phase of the cell cycle and is highly expressed in various cancers including bile duct [18], colon [10], liver [15], and lung [29] cancers. In the current study, MPS1 was significantly up-regulated in hyperplasia/dysplasia (precancerous lesions) and CCA in a hamster CCA model at all time points examined. MPS1 expression could be detected in precancerous lesions as early as 1-month after Ov infection. These findings suggested that MPS1 may be involved in cholangiocarcinogenesis. This is the first report revealing the involvement of MPS1 in the carcinogenic process of CCA in the hamster model. Overexpression of MPS1 was also demonstrated in large panels of CCA cases retrieved from two public databases and the tissue microarray of 185 CCA patient tissues and was significantly correlated with shorter survival of CCA patients. MPS1 was an independent factor for poor prognosis of CCA patients. These data strongly suggest that MPS1 is a mitotic kinase protein that may play important roles in CCA. These observations agree with previous reports in pancreatic [13], breast [30], and liver [31] cancers. 

There were multiple roles for MPS1 in cell proliferation, colony formation, migration, and invasion of CCA cells demonstrated in the current study. Suppression of MPS1 by siRNA inhibited cell proliferation and colony formation ability in CCA cell lines, which was similar to observations in liver cancer [15,31,32] and medulloblastoma [33]. MPS1 generates the mitotic checkpoint complex, which control chromosome segregation in mitosis [5,6,7]. Therefore, suppression of MPS1 may result in chromosome segregation errors which could subsequently induce G2/M arrest. Cell cycle analysis showed that knockdown of MPS1 remarkably induced G2/M arrest and increased sub-G1 (apoptotic cell) populations in KKU-055 and KKU-213A CCA cell lines. These data correspond to previous studies in colon cancer [10], endometrial cancer [34] and medulloblastoma [10,33].

The involvement of MPS1 in the progression of CCA was explored. Knockdown of MPS1 significantly suppressed cell migration and invasion in KKU-055 and KKU-213A cell lines. These findings are similar to observations reported in bladder [14], breast [17], liver [15,31], and lung [16,35] cancers. Epithelial-mesenchymal transition (EMT) is a process involved in the migration and invasion of cancer cells. Decreasing expression of epithelial markers (claudin-1, E-cadherin, and ZO-1) and increasing expression of mesenchymal markers (N-cadherin, slug, and vimentin) are the most common changes associated with EMT. The EMT is regulated by several signaling pathways including the PI3K/AKT, JAK/STAT, and MAPK signaling pathways [36,37]. Previous studies have reported that MPS1 enhanced cell migration and mediated EMT via AKT activation in several cancers [14,17,31]. The alteration of EMT-related marker expression and signaling pathways in CCA cells by MPS1 was investigated. Suppression of MPS1 significantly increased the expression of claudin-1 but decreased the expression of slug and vimentin in KKU-055 and KKU-213A cells. This result is consistent with previous studies showing that vimentin expression was decreased in stable knockdowns of MPS1 in triple negative breast cancer [17] and lung cancer [35]. Nevertheless, this current research is the first to demonstrate an increase in claudin-1 and decrease in slug expression after MPS1 knockdown in CCA cells.

To explore whether the AKT and STAT3 signaling pathways were modulated by MPS1, CCA cells were pre-treated with siMPS1 followed by an AKT inhibitor (MK2206), an AKT activator (SC79), or a STAT3 inhibitor (Stattic). The expression of EMT related markers were examined by Western blot. Knockdown of MPS1 or MK2206 treatment significantly reduced phosphorylation of AKT (pAKT) and altered expression of the EMT related markers in both CCA cell lines. Combination of siMPS1 and MK2206 further reduced pAKT and altered the expression of EMT-related markers. This finding supports a previous study that displayed activation of AKT in an MPS1 overexpressing hepatocellular carcinoma cell line [31]. Co-treatment with siMPS1 and SC79 could recover pAKT and restore expression of EMT markers. Altogether, this strongly confirms that MPS1 regulated EMT via the AKT signaling pathway in CCA cells. We also demonstrated that MPS1 may regulate EMT via the STAT3 pathway. Silencing MPS1 significantly suppressed pSTAT3 and altered expression of the EMT markers. Combination of siMPS1 and Stattic treatment further diminished pSTAT3 and affected the expression of EMT markers in CCA cells. This is the first report demonstrating that MPS1 regulates EMT via STAT3 signal transduction. The correlation of MPS1 expression and pAKT or pSTAT3 in hamster and human CCA tissues is interesting for further study.

Depletion of MPS1 by siRNA remarkably alleviated invasion of CCA cell lines. These data are similar to numerous publications in other cancers [14,16,17,31]. The gelatin zymography exhibited that suppression of MPS1 significantly reduced MMP-2 and MMP-9 activities in both KKU-055 and KKU-213A, which are related to an invasive phenotype. This was the consequence of STAT3 activation, which regulates the expression of MMP-2/9 [38,39]. Our results demonstrate that MPS1 controls the MMPs-dependent invasion of cancer cells. Apart from its effect on AKT and STAT3 signal transduction, MPS1 may modulate a set of proteins that play roles in the progression of CCA. Our future studies will focus on the proteomic profile of MPS1-mediated progression of CCA. Concerning the metastatic role of MPS1 in animal model, no metastasis was observed in the hamster model. This may suggest the study of a tail vein metastasis model, which involves the injection of MPS1 knockdown cells via tail vein and analysis of their ability to form tumors and/or to colonize at distant sites e.g., lung via hematogenous spread. Moreover, it is interesting to study the spheres formation after MPS1 knockdown. This may lead to address the question of MPS1 function in cancer stem cell of CCA. 

Many MPS1 inhibitors have been investigated as cancer therapeutics in experimental, preclinical, and clinical studies [34]. We have determined the effect of one MPS1 inhibitor, reversine, on CCA cell lines [40]. This would demonstrate the potential value of targeting MPS1 in the treatment of CCA. More preclinical studies on the inhibition of MPS1 functions using specific inhibitors or MPS1 knockdown/overexpression in CCA are required.

**Figure 6:**
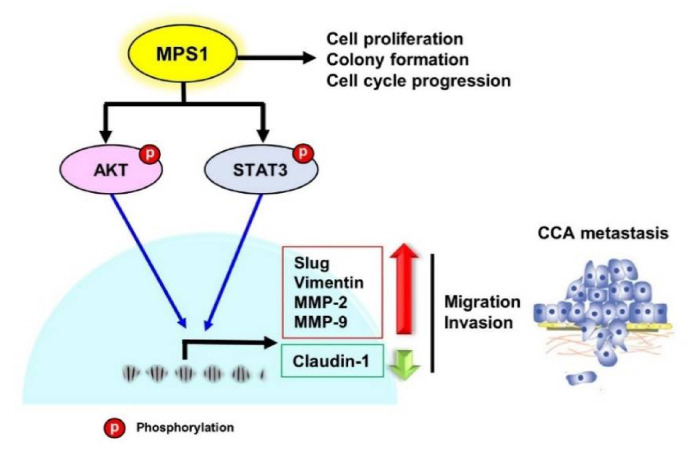
The proposed molecular mechanism of how MPS1 promotes the metastasis of CCA. MPS1 contributes to cell proliferation, colony formation, and alters cell cycle progression. MPS1 activates AKT and STAT3 signal transduction. These actions consequently enhance the expression of EMT markers slug, vimentin, and MMP-2/9, while modulating expression of claudin-1. These result in an increase in migration and invasion, which lead to metastasis in CCA.

Collectively, our research clearly suggests that MPS1 could be a promising prognostic marker for CCA patients. Multifunctional MPS1 regulates cell proliferation, colony formation, cell cycle progression, migration, and invasion of CCA cells and enhances EMT and MMPs-mediated metastasis via activation of AKT and STAT3 signaling pathways in CCA (Figure 6).

## 5. Conclusions

Our research highlighted that MPS1 contributes to cholangiocarcinogenesis and CCA progression. MPS1 could serve as a promising prognostic marker for CCA patients. MPS1 promoted cell proliferation and EMT-related migration via AKT and STAT3 signaling pathways and MMPs-dependent invasion of CCA cell lines. These findings indicate that MPS1 could be an effective target for treating CCA metastasis.

## Figures and Tables

**Table 1:** MPS1 expression in Ov-associated CCA in hamster model.

Treatment Group	1 M			3 M			6 M		
	NBD	HP/DP	CCA	NBD	HP/DP	CCA	NBD	HP/DP	CCA
Control	0/4 (0)	-	-	0/5 (0)	-	-	0/5 (0)	-	-
Ov	0/4 (0)	2/4 (50)	-	0/4 (0)	3/4 (75)	-	-	4/4 (100)	-
NDMA	0/4 (0)	-	-	0/4 (0)	1/3 (33)	-	0/4 (0)	3/4 (75)	-
Ov + NDMA	0/4 (0)	2/4 (50)	-	0/2 (0)	5/5 (100)	5/5 (100)	-	5/5 (100)	5/5 (100)

All data are expressed as the number of MPS1 positive cases/total cases with the histological classification. The numbers in parentheses are the percent of MPS1 positive cases. Ov: *Opisthorchis viverrini*-infected group, NDMA: N-Nitrosodimethylamine-treated group, NBD: normal bile duct, HP/DP: hyperplasia/ dysplasia, CCA: cholangiocarcinoma and (-) not detected.

**Table 2:** Multivariate analysis of MPS1 expression in 185 CCA patients.

Characteristics (*n*)	Univariate Analysis *p*-Value	Multivariate Analysis		
		HR	95% CI	*p-*Value
Gender (185) (ref. group Female)	0.271			
Age (183) (ref. group < 57)	0.194			
CCA type (185) (ref. extrahepatic CCA)	0.883			
Histological Type (177) (ref. group Papillary)	0.276			
Lymph nodemetastases (168) (ref. group N0)	< 0.001	1.985	1.346–2.928	0.001
Tumor size (183) (ref. group < 7)	0.020	1.577	1.033–2.408	0.035
Tumor stage (175) (ref. group I-III) IVA	0.016	1.691	1.115–2.565	0.013
IVB	0.001	3.702	1.901–7.208	< 0.001
MPS1 expression (185) (ref. group MPS1 Negative)	< 0.001	2.015	1.442–3.073	< 0.001

## Data Availability

All data generated or analyzed during this study are included in this published article.

## Supplementary Materials

The following are available online at https://www.mdpi.com/2227-9059/9/1/68/s1, Table S1: MPS1 expression and clinicopathological features of CCA patients from GEPIA and GEO databases. Table S2: MPS1 expression and clinicopathological features of 185 CCA patients, Figure S1: MPS1 protein expression in four CCA cell lines. Figure S2: The expression of Cyclin B1, Mcl-1 and Bax after MPS1 knockdown in CCA cell lines.
