# Supplementary material for: High Monopolar Spindle 1 Is Associated with Short Survival of Cholangiocarcinoma Patients and Enhances the Progression Via AKT and STAT3 Signaling Pathways
Source: Biomedicines. 2021 Jan 13;9(1):68. doi: 10.3390/biomedicines9010068 (PMC7828338; doi:10.3390/biomedicines9010068)
Supplement: Supplementary file 1 [file biomedicines-09-00068-s001.pdf]

Supplementary Table S1 MPS1 expression and clinicopathological features of CCA patients from GEPIA and GEO databases

**Characteristics**	**GEPIA (TCGA-CHOL)**				**GEO (GSE89749)**			
	**MPS1 expression**				**MPS1 expression**			
	**No. of patient**	**Negative (<7)**	**Positive (≥7)**	***P*-value**	**No. of patient**	**Negative (<7.8)**	**Positive (≥7.8)**	***P*-value**
**Gender**	36				91			
Female	20	8	12	0.179	42	19	23	0.722
Male	16	10	6		49	24	25	
**Age**	36				91			
<57	18	9	9	0.999	42	20	22	0.948
≥57	18	9	9		49	23	26	
**Tumor type**					91			
Intrahepatic		N/A	N/A		63	35	28	**0.017**
Extrahepatic		N/A	N/A		28	8	20	
**Histological type**					87			
Papillary		N/A	N/A		19	6	13	0.099
Non-Papillary		N/A	N/A		68	36	32	
**Ov infection**					91			
Positive		N/A	N/A		43	12	31	**<0.001**
Negative		N/A	N/A		48	31	17	
**Lymph node invasion (N)**	31				81			
N0	26	14	12	0.333	56	26	30	0.59
N1	5	1	4		25	10	15	
**T stage**	36				91			
T1-T2	31	16	15	0.629	33	16	17	0.178
T3-T4	5	2	3		58	27	31	
**M stage**	33				90			
M0	28	15	13	0.576	85	41	44	0.72
M1	5	2	3		5	2	3	
**Pathological Staging**	36				84			
I-III	29	16	13	0.285	52	25	27	0.94
IVA	2	0	2		27	13	14	
IVB	5	2	3		5	2	3	

N/A = not available

Supplementary Table S2 MPS1 expression and clinicopathological features of 185 CCA patients

Characteristics	MPS1 expression			
	No. of patient	Negative (H-score = 0)	Positive (H-score > 0)	*P*-value
**Gender**	185			
Female	62	40	22	0.496
Male	123	73	50	
**Age**	183			
<57	83	47	36	0.247
≥57	100	65	35	
**Tumor type**	185			
Intrahepatic CCA (iCCA)	175	103	72	0.320
Extrahepatic CCA (eCCA)	10	8	2	
**Histological type**	177			
Papillary	88	51	37	0.406
Non-Papillary	89	57	32	
**Lymph node invasion (N)**	168			
N0	83	54	29	0.405
N1	85	50	35	
**Tumor size (cm)**	185			
<7 cm	132	77	55	0.301
≥7 cm	51	34	17	
**Tumor stage**	185			
I-III	117	77	40	0.113
IVA	53	30	23	
IVB	15	6	9	

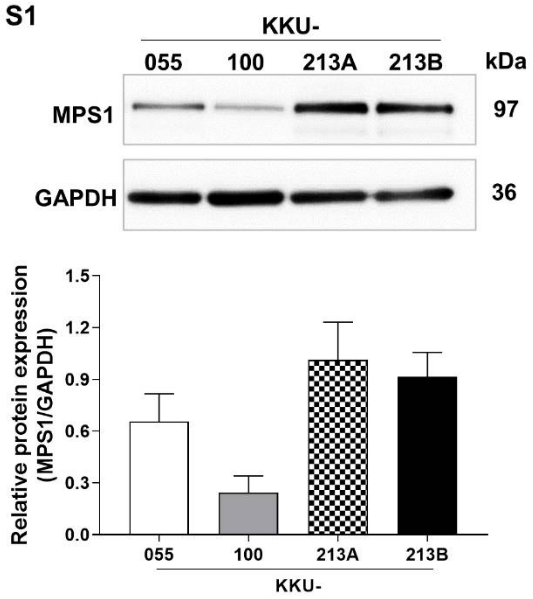

Supplementary Figure S1. MPS1 protein expression in four CCA cell lines. Western blot result shows MPS1 protein expression in KKU-055, KKU-100, KKU-213A and KKU-213B CCA cell lines. Bar graph represents the relative protein expression of MPS1 which was normalized to GAPDH.

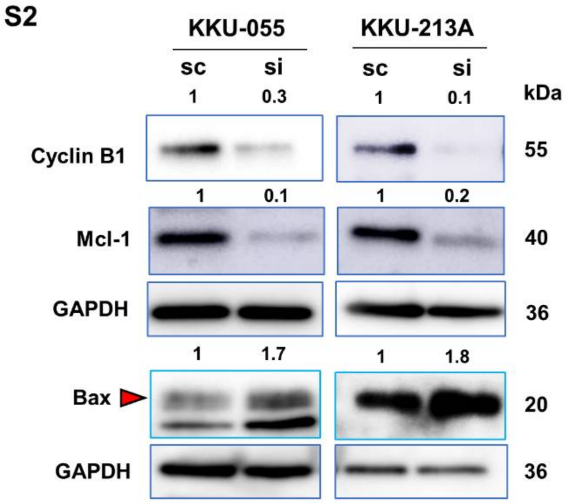

Supplementary Figure S2. The expression of Cyclin B1, Mcl-1 and Bax after MPS1 knockdown in CCA cell lines. The protein expression was normalized to GAPDH. The relative fold change to scramble control (sc) was quantified and indicated by the numbers above the corresponding panels.
